# Enhancing recovery: surgical techniques and rehabilitation strategies after direct anterior hip arthroplasty

**DOI:** 10.1186/s10195-024-00786-y

**Published:** 2024-09-30

**Authors:** Alberto Di Martino, Christopher Keating, Michael J. Butsick, Daniela Platano, Lisa Berti, Louis N. Hunter, Cesare Faldini

**Affiliations:** 1 1st Orthopedic and Traumatology Clinic, IRCCS Rizzoli Orthopedic Institute, Via Giulio Cesare Pupilli 1, 40136 Bologna, Italy; 2 Department of Biomedical and Neuromotor Science—DIBINEM, University of Bologna, 40136 Bologna, Italy; 3 Department of Physical Therapy, Jefferson College of Rehabilitation Sciences, Thomas Jefferson University, Philadelphia, PA United States; 4 Physical Medicine and Rehabilitation Unit, IRCCS Istituto Ortopedico Rizzoli, Bologna, Italy; 5 Sidney Kimmel Medical College, Thomas Jefferson University, Philadelphia, PA, United States; 6 Jefferson College of Health Professions, Thomas Jefferson University, Philadelphia, PA, United States

**Keywords:** Total hip arthroplasty, Direct anterior approach, Rehabilitation

## Abstract

Total hip arthroplasty (THA) is a common surgical procedure for hip joint pathologies, with the direct anterior approach (DAA) gaining popularity due to potential benefits in postoperative recovery. This review aims to provide a comprehensive analysis of rehabilitation strategies following DAA THA, focusing on surgical techniques, postoperative care, and outcomes. The evolution of the DAA to THA is discussed, highlighting historical advancements and comparisons with other surgical approaches. Surgical techniques and considerations specific to the DAA are detailed, including outcomes and complications compared to alternative approaches. The role of the surgical technique in influencing postoperative rehabilitation is explored, emphasizing the importance of optimizing surgical procedures for enhanced recovery. Postoperative care and rehabilitation models following DAA THA are examined, with a focus on the impacts of different rehabilitation protocols on patient outcomes. The review underscores the significance of tailored rehabilitation programs in promoting optimal recovery and patient satisfaction. Current evidence from recent studies, meta-analyses, and clinical trials is critically analyzed to provide insights into the effectiveness of postoperative rehabilitation strategies. The review identifies gaps in the existing literature and proposes recommendations for future research to improve rehabilitation protocols and enhance outcomes. In conclusion, this review highlights the importance of postoperative rehabilitation in the context of DAA THA. By synthesizing historical perspectives, current evidence, and future directions, the review offers a comprehensive understanding of rehabilitation strategies following DAA THA. The findings underscore the need for personalized rehabilitation programs and ongoing research to optimize postoperative recovery and improve outcomes in the field of THA.

## Introduction

Annual incidence rates of total hip arthroplasty (THA) surgeries are projected to increase in the US to 635,000 by 2030 [1]. Researchers have attempted to prepare for this by identifying the most cost-effective surgical and postoperative strategies [2]. One THA approach receiving considerable attention is the direct anterior approach (DAA) initially introduced by Heuter in 1881 [3]. The DAA is proposed to have the benefits of neuromuscular sparing, earlier discharge timelines, reduced risks of dislocations, reduced pain and opioid utilization, and faster postoperative recovery [2, 4–6]. However, there is conflicting evidence regarding some of these results when the DAA is compared to other surgical techniques, and there are concerns about its high overall costs ($280,000 per case) [2, 7]. As reimbursement rates and quality outcomes continue to be scrutinized by hospitals, insurance companies, and government stakeholders, the financial burden and postoperative outcomes of the DAA have been questioned [8].

The improved short-term outcomes in those with DAA THA dissipate in comparison to other surgical approaches, with a limited difference in physical activity at and beyond 6 months [9, 10]. Pain is reported to be better controlled in the DAA; however, narcotics continue to be the primary pain strategy prescribed for THA despite an identified opioid epidemic [11]. These outcomes raise the question of which postoperative approaches are being applied after DAA THA.

Research is lacking in relation to what the postoperative recovery should entail and how to deliver the rehabilitation model in terms of timing, frequency, intensity, and specific treatments for various patient populations that have undergone a THA. Fast-track models have been implemented and studied as one type of model that is proposed to allow an earlier recovery [12]. The ongoing uncertainty in the literature due to various self-developed surgical protocols and precautions contributes to the clinical question of the effectiveness of rehabilitation programs for individuals after DAA THA [13].

Important factors that have been proposed for selecting postoperative physiotherapy management rather than relying on self-guidance include initial pain levels and prior reported level of function [13–15]. These rehabilitation decisions come with the controversial decision to impose postoperative patient restrictions [16]. A recent meta-analysis identified that only 22% of patients abide by their prescribed surgical precautions, noting that increased patient satisfaction, better sleep, and earlier returns to independent ambulation occurred in those who did not abide by these postoperative restrictions [17]. There is much to investigate regarding the postoperative management of the DAA in THA, including discharge plans, pain management, and rehabilitation models, in order to enhance long-term recovery with an overall goal to reduce the healthcare burden. The purpose of this article is to review the best available DAA THA surgical techniques and the current evidence for postoperative rehabilitation.

In this review, we aim to provide a comprehensive analysis of the current landscape of rehabilitation following DAA THA. We will delve into key considerations such as surgical techniques, postoperative care strategies, and the impact of rehabilitation on patient outcomes. The review will critically evaluate recent studies and meta-analyses to offer insights into the effectiveness of different postoperative rehabilitation approaches. Furthermore, we will discuss the importance of tailored rehabilitation protocols in optimizing recovery and enhancing patient satisfaction. We will first explore the evolution of the anterior approach to hip arthroplasty, which will be followed by an in-depth discussion of rehabilitation models, outcomes, and future research directions. By the end of this review, healthcare providers and researchers will gain a comprehensive understanding of the current evidence for and challenges in rehabilitation after DAA THA.

## Evolution and current standards

Carl Hueter described the possibility of using the interval between the tensor fasciae latae and sartorius muscle to access the hip joint in 1817 [18]. At that time, the anterior approach was used to manage war injuries and for the treatment of infectious diseases of the femoral head such as coxitis. Smith-Petersen popularized the same approach throughout the English-speaking scientific community after describing its use for the open reduction of congenital dislocation of the hip in 1917. After the introduction of THA implants, many surgeons attempted to use the intermuscular Smith-Petersen approach to access the hip joint and minimize THA surgical trauma. The modern anterior minimally invasive surgical approach (AMIS; Medacta, Switzerland) was developed by F. Laude in 1990 in Paris [19]. This technique was brought to the United States by Matta, who subsequently further modified it [20]. The procedure required the use of a traction table and dedicated instrumentation with curved retractors, offset handles for acetabular reaming and cup implantation, and curved handles for femoral preparation and stem implantation. The evolution in instrumentation occurred in parallel to the evolution of the design of implants. Laude popularized a shorter corail-type stem that allows easier implantation via the DAA due to the decreased size of the shoulder of the implant, which has a modified surface coating [21, 22]. Continued modification of DAA THA was described in relation to its use in various patient populations, including severe dysplastic, elderly, and obese patients, all of whom were once considered less suitable for the AMIS approach [23–29].

Once the patient is eligible for a THA implant, proper planning is performed to check the size and position of the prosthetic implant, check that the patient’s deformity is adequately managed with the surgery, and check that the DAA is the most suitable approach to address it [30]. The surgical technique used for DAA THA has significantly evolved in the last 20 years. At present, most implant companies provide dedicated instrumentation and implants to ease the trauma from surgery and to limit the soft-tissue exposure. Surgery is performed in a supine position, and it can be performed on a standard radiolucent table that allows the hips to be extended, or on a dedicated traction table; the use of the traction table is associated with specific pros and cons [31]. The main issue with the use of the traction table is the impossibility to directly assess, during surgery, the length discrepancy between limbs. Conversely, on a standard table, there is the need for one more surgical assistant, and a more extended soft-tissue dissection is usually required to gain sufficient exposure to the hip joint, especially for femoral exposure.

**Fig. 1:**
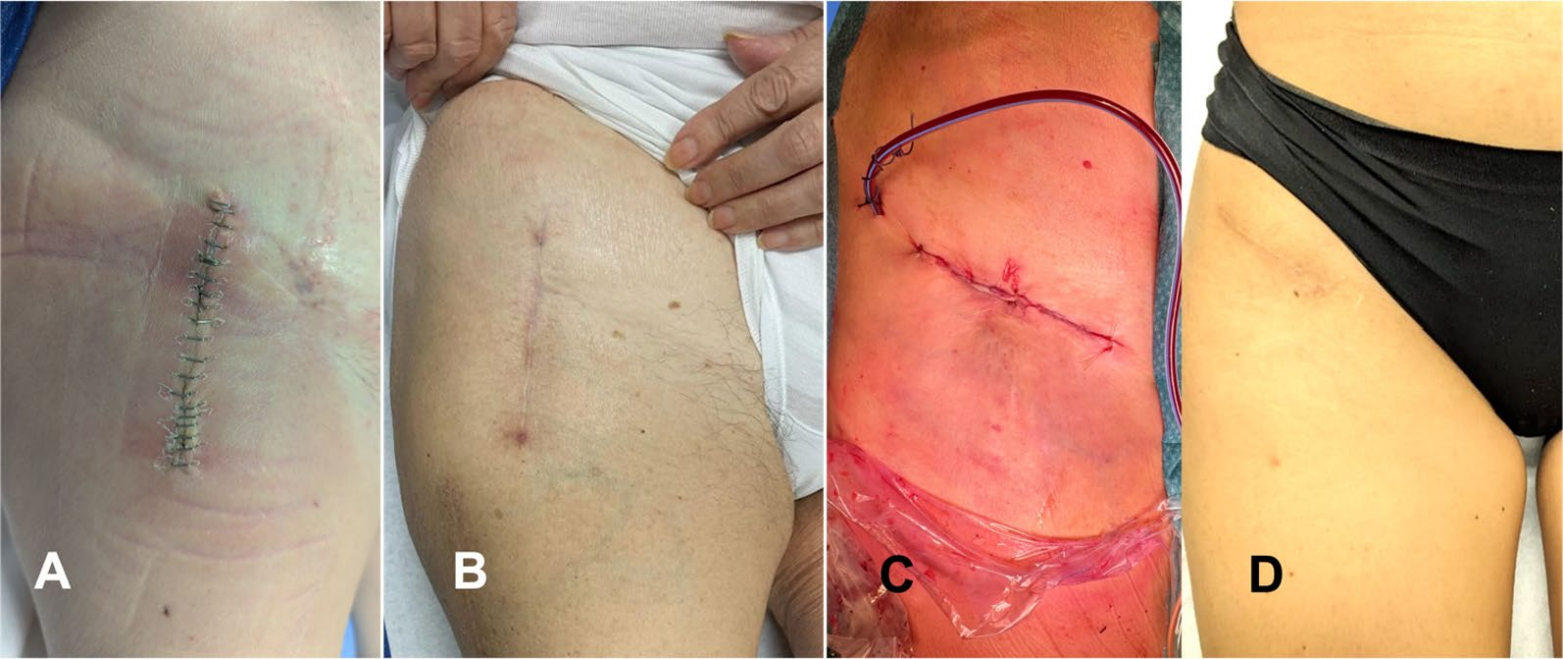
The standard longitudinal incision (**A** and **B**) and bikini incision (**C** and **D**) used in the DAA. **A** One-week-postoperative suture of a standard incision with metallic clips. **B** Normally healed at the 6-month follow-up. **C** Intraoperative photograph of a bikini incision sutured with absorbable stiches. **D** Normally healed at the 6-month follow-up

The traction table requires the foot of the operated leg to be restrained by a boot, and limb movements are supervised by an assistant to control the hip traction, flexion, rotation, adduction, and abduction. The hip is positioned with 10° of internal rotation and slight abduction, and approximately 15° of hip flexion is maintained using a step. The surgical incision is usually performed according to one of two techniques: the standard longitudinal and the bikini incision (Fig. 1).

**Fig. 2:**
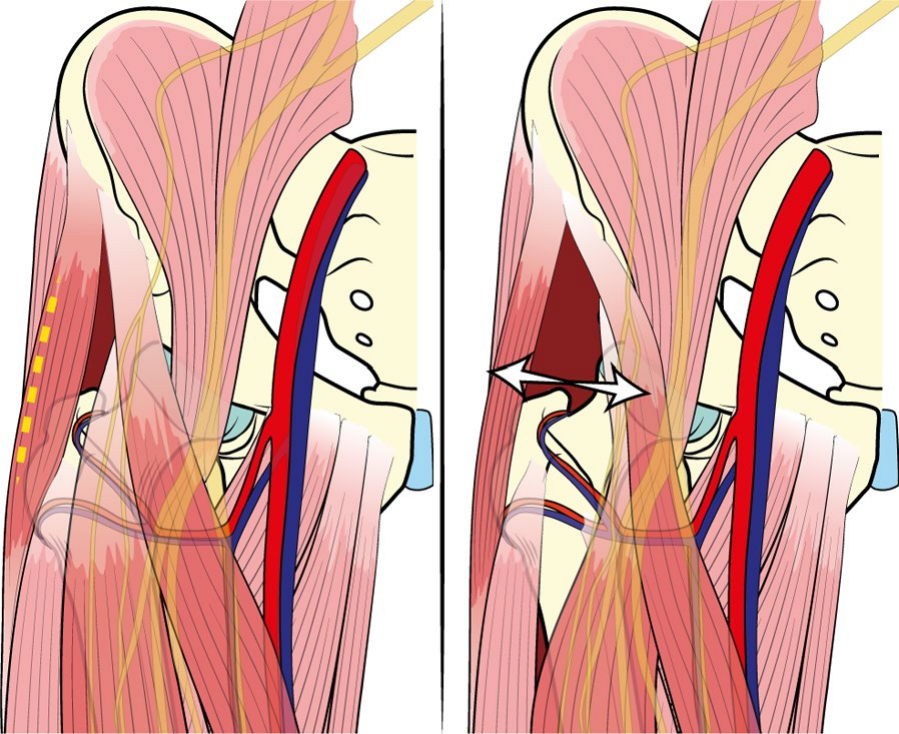
Anatomical and surgical associations. The incision is performed over the tensor fasciae latae muscle belly (*left picture*), and access to the hip joint is later achieved by separating the tensor fasciae latae and sartorius muscle (*arrows*). The lateral femorocutaneous nerve and arterial rami of the deep circumflex artery are encountered during access

**Fig. 3:**
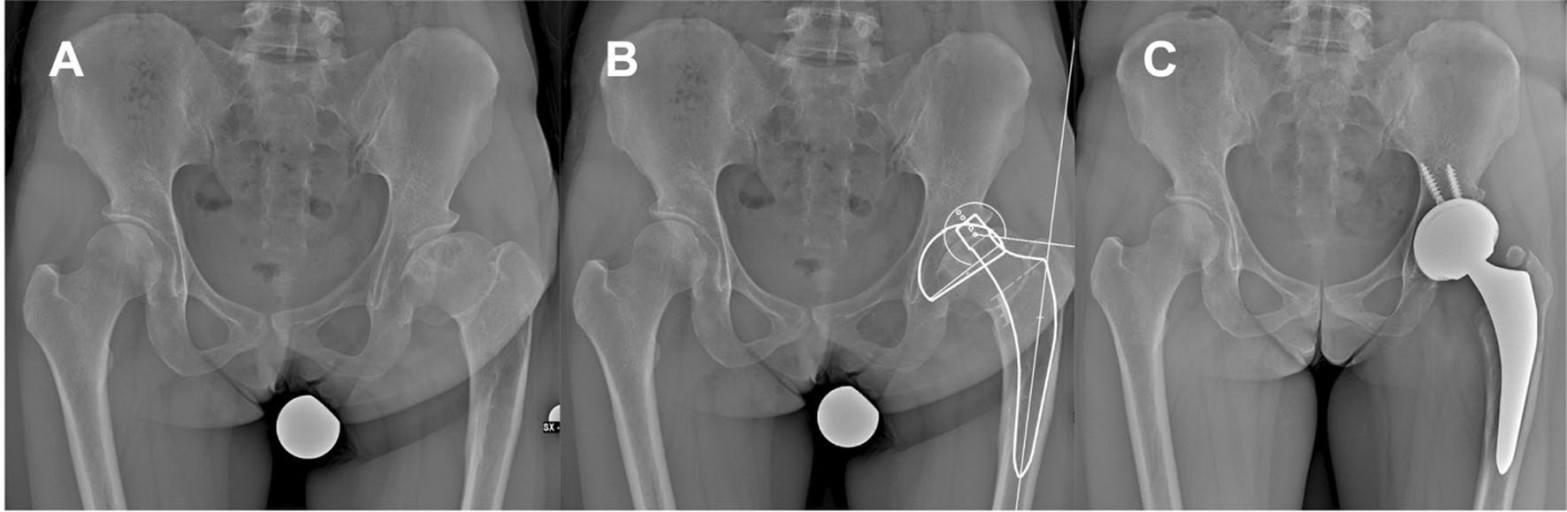
Secondary arthritis of the left hip after Legg–Calvé–Perthes disease (**A**). Preoperative digital templating (**B**). THA surgery was performed through the minimally invasive DAA (**C**)

The standard incision begins 2 cm distally and 2 cm laterally from the anterior superior iliac spine and is extended distally for approximately 8 cm. The bikini incision has approximately the same length and follows the Langer lines, promoting a more aesthetic outcome. The bikini incision is typically performed at the inguinal fold, aligning obliquely from lateral to medial and from proximal to distal [24]. Subcutaneous plane dissection exposes the fascia of the tensor fascia latae muscle, which is incised longitudinally and slightly laterally over the muscle belly to avoid damage to the lateral femoral cutaneous nerve (LFCN), which runs along the sartorius muscle in most patients. The LFCN has three main location variants and sometimes crosses the surgical field, requiring intraoperative isolation and exposure [32]. It can be injured during surgery or retraction when it is in its usual position along the sartorius muscle as well, resulting in numbness at the anterior aspect of the thigh (Fig. 2). After surgery, careful hemostasis control and positioning for drainage may be required if there is a risk of postoperative bleeding [33, 34, 48]. A final X-ray check is performed at the end of surgery to check the implant positioning (Fig. 3).

### Surgical outcomes

THA is a widely used surgical intervention for end-stage degenerative hip osteoarthritis and displaced femoral-neck fractures that provides pain relief, functional restoration, and improved quality of life [35–37, 63]. Given the popularity of the minimally invasive DAA, a large amount of literature focusing on the advantages and complications of the DAA over conventional techniques has been produced. There is no clear consensus on the best surgical approach because of study heterogeneity. Research involving different surgical comparisons (the DAA, the conventional approach, the direct lateral approach (LA), the posterior-lateral approach (PLA), or the posterior approach (PA)), the use of different outcome measures, and the presence of various levels of study quality contribute to this heterogeneity [61].

The minimally invasive surgical direct anterior approach (MiS-DAA) for THA is considered advantageous over conventional techniques because of reduced muscular damage [38] as indicated by lower levels of the muscle damage marker creatine kinase [39], reduced blood loss, and diminished postoperative pain. According to a recent systematic review with a meta-analysis, DAA THA resulted in significant reductions in the length of hospitalization [40–42], rate of dislocation [41, 42], and overall postsurgical complication rate [42] (see Table 1). However, when only randomized clinical trials were considered in the meta-analysis, no statistically significant difference in terms of risk of dislocation, periprosthetic fracture, or venous thromboembolism was found when the DAA was compared with the PLA or LA, and no significant difference in risk of neurapraxia between the DAA and the LA was reported [40]. Several network meta-analyses have compared the results of the DAA with other approaches in recent years. While poorer outcomes were reported in those patients operated on by LA, in qhich gluteus medius sacrifice was required, differences between the results of the DDA THA and the results of THA achieved through other minimally invasive approaches were less evident.

**Table 1:** Surgical outcomes, pain and functional outcomes of the DAA vs conventional techniques

**Surgical outcome**	
Reduced muscular damage [40, 41]	
Reduced length of hospitalization [42–44]	
Reduced overall postsurgical complication rate^#^ [44]	
**Pain reduction**	
In the very short term	Within hours [51, 59] or on 1st PO day [54]
Within a few days	Day 1 [4, 53], day 1–2 [58], day 1–3 [39, 56, 60], day 1–4 [6, 50, 52], day 3 [59], day 5 [55], day 1–6 [49]
Within weeks	Within 1 week [55]
	Within 2 weeks [64]
	At 1–3 and 6 weeks [50]
	At 2 weeks (but not at 6 weeks) [56]
	At > 16 days (but not at 1, 5, and 16 PO days) [65]
After 1 month but < 3 months	At 1 month [62] and 40 PO days [65]
Within 3–6 months	No difference [62]
**Functional outcome**	
Improved HHS	At 6 weeks (vs PA group) [42]
	At 12 weeks (vs LA group) [42]
	At 6 months (vs PA group) [80]
	At 1 year (vs LA group) [81]
Improved HOOS	At 3 months (vs LA in the symptoms/stiffness subscale) [4]
Improved WOMAC	At 3 weeks (vs PA group) [55]
	At 6 weeks (vs LA group) [82]
	At 1 year (vs LA group) [81]
Improved physical activity (UCLA Activity Score)	At 3 months (vs LA group) [41]
Improved T25-FW	At 6 and 12 months (vs LA group) [83]

^#^Not reported when only randomized clinical trials were considered for meta-analysis [42]

*PO* postoperative,* LA* lateral approach,* PA* posterior approach,* PLA* posterior-lateral approach,* HHS* Harris Hip Score,* HOOS* Hip Disability and Osteoarthritis Outcome Score,* WOMAC* Western Ontario and McMaster University Osteoarthritis Index,* UCLA* University of California at Los Angeles,* T25-FW* timed 25-m foot walk

Yan et al. [43] used quantitative outcomes to compare the different approaches to THA in randomized clinical trials (RCTs). They analyzed data from 63 studies investigating 4,859 patients with a median follow-up time of 1 year, comparing eight different surgical approaches to THA in terms of performance. No difference was found when comparing the DAA and Postero-Lateral (PL) approaches, but the PL approach required less surgical time. In a more recent meta-analysis by Ang et al. [40] that compared 24 studies comprising 2,010 patients, they found that the DAA was positively associated with improved early functional outcomes and a shorter mean length of stay, but it was also associated with a longer operative time than the PL approach. In the meta-analysis of 11 RCTs by Yang et al. [44], which reported on 932 patients who underwent THA by the DAA or PL approach, it was found that the DAA gave comparable results to the PL in terms of functional recovery. The DAA allowed earlier discontinuation of the use of walking aids, led to lower postoperative pain scores, and led to better cup positioning, even though it was associated with a higher incidence of LFCN injury.

When the DAA and superpath approaches were compared, no difference in early functional outcomes or cup positioning was found [45]. Gluteus medius preservation and the surgeon’s preference and skill in the determined surgical approach seem to be the major determinants of overall patient outcomes after THA. An accurate training curve is required for minimally invasive THA surgery performance because the techniques are usually more demanding and minimally invasive THA surgery might expose patients to complications and increase the overall costs for the health system [79]. The DAA does offer faster recovery and muscle integrity preservation, speeding up the recovery of patient independence and autonomy [2].

### Pain outcomes

Studies on postoperative pain and function describe an overall superiority of the DAA compared to other techniques, with positive results, particularly in the early postoperative stages. A faster recovery postoperatively can be attributed to improved pain control both at rest and during mobility. This is due to the muscle-sparing surgical approach and early weight bearing, which promotes joint lubrication, improves circulation, and prevents perceived stiffness. In the case of THA after fracture in the elderly, acute postoperative pain intensity is predictive of recovery time to independent walking [46]. As a result, strategies for improved pain control to promote earlier functional outcomes are considered during surgical technique selection as well as rehabilitation prescription.

The use of DAA THA has had mixed results when pain was the key outcome. Several studies have found that pain after DAA THA, when compared to other approaches, was not significantly better [64–66]. More recent work has determined that the DAA does lead to a significant improvement in pain outcomes after surgery [6, 50, 67, 68]. Nistor et al. identified myoglobin as a possible confounder of pain reports from patients. The presence of this known muscle damage molecule can be more strongly associated with pain reports than the type of surgical approach used [6]. The DAA limits the amount of muscle damage from the hip external rotators and may contribute to improved pain reports in the short term as well as improved mobility in the acute setting [53, 66, 69, 70]. DAA THA allows for greater ambulation immediately postoperatively and for the first 4 weeks. There does not appear to be a difference between techniques after that period, but that could be impacted more by the type of rehabilitation received than the surgery itself. The ambulation, stair-climbing, transfer training, balance, and mobility interventions provided by rehabilitation professionals in the acute phase have a positive impact on pain reports and mobility based on the above evidence as well as case reports [71–73].

### Functional outcomes

Different measures have been used for global and ambulation function in assessments at various time points. Several tools are available to evaluate the functional outcomes of THA according to various measurement settings [74]. Patient-reported outcome measures (PROMs) are self-reported questionnaires that assess functional ability and quality of life from the patient's perspective; these include the Harris Hip Score (HHS) and the Hip Disability and Osteoarthritis Outcome Score (HOOS), health-related quality of life (QoL) questionnaires, the Western Ontario and McMaster University Osteoarthritis Index (WOMAC), the Medical Outcomes Study Short Form 36 (SF36), and the UCLA (University of California at Los Angeles) Activity Score.

Since PROMs are subjective evaluations, they are prone to individual and psychological bias and should not be used alone, especially in the first phases after THA, when patients might underestimate or overestimate their functional ability in relation to pain perception [75]. Amid the heterogeneous results on outcome indicators and timing of data collection in the literature, the meta-analysis by Ang and colleagues [40] reported a significantly higher HHS (i.e., lower pain and better function [76]) 6 or 12 weeks after surgery in patients who underwent DAA THA when compared to those in whom the PA or LA was used, respectively. As shown in Table 1, the HHS was significantly higher in DAA patients at 6 and 12 weeks [40], 6 months [77], and 1 year [78] when compared to a conventional technique. However, for longer follow-ups of 5 years [51] or further [4], no superiority of either technique was found. Other PROMs after the DAA approach are reported in Table 1. One study reported a better outcome of the DAA group for the physical subset of the SF36 questionnaire at 3 weeks and 3, 6, and 12 months when compared to the LA group.

Performance-based tests (PBTs) allow the objective measurement of functional movement. In the case of lower-limb surgery, mobility performance, balance, and postural control can be assessed. PBTs, including the timed 25-m foot walk (T25-FW), 10-m walk test (10TMWT), timed up and go test (TUG), and stair-climbing test (SCT), can quantify performance through tasks mimicking activities of daily living. Outcome data from PBTs are shown in Table 1. Moreover, biomechanical examinations (hip strength, gait, and balance) provide objective quantitative parameters which correlate to functional performance. Finally, to cover the three domains of the World Health Organization’s International Classification of Functioning, Disability and Health (https://icd.who.int/dev11/l-icf/en), other scores such as the Barthel Index, the Modified Rankin Scale, and the Functional Independence Measure (FIM) are used to evaluate activity and participation.

A wide variety of parameters are reported in PBTs, including the number of feet ambulated [81, 82], the walking distance, the achievement of unaided walking [52, 55, 83], independent mobilization with walking aids (single-point cane, crutches, or a rolling walker) [52, 55, 84–86], the ambulatory decline [81], the achievement of full weight-bearing on the injured leg [86], and the degree of mobilization [52]. An altered gait pattern such as a Trendelenburg gait is another indicator of functional outcome [47, 56, 84]. As a measure of participation, the Barthel Index [52, 62], the Katz index of activities of daily living (ADL) [59], FIM [55], or returning to daily activities (return to driving, return to work) [55, 87] can also be used for comprehensive assessment.

### Considerations regarding the timing and functional advantages of the DAA

1. The DAA approach leads to lower pain levels in the early follow-up period than the PL approach.
2. The advantage in postoperative recovery appears to be short-lived in most cases. The increase in functional scores associated with the DAA is maintained up to 4–12 weeks after surgery in most studies. Nevertheless, this result is advantageous for a better functional recovery during the early postoperative phase; to prevent the rapid progression of disuse atrophy, especially in the elderly; and to speed up outpatient rehabilitation. By promoting functional recovery immediately after surgery, the DAA may improve subsequent hip joint function.

## Postoperative care and rehabilitation models

Rehabilitation is an essential part of the process to ensure optimal outcomes. The rehabilitation process consists of distinct phases that start before surgery, with patient education on precautions and contraindications specific to the surgical procedure, and continue until the complete or satisfactory recovery of function. The physiotherapy program should always be individualized based on the patient’s specific needs, preoperative conditions, eventual postoperative complications, and functional goals. Close communication between the surgical team, the physiotherapist, and the patient is essential to tailor the rehabilitation program effectively and to achieve a full functional recovery.

### Initial rehabilitation

The first phase of rehabilitation is aimed at regaining independence in mobility (with or without an assistive device), self-care, and exercises to improve strength and range-of-motion impairments, ensuring joint protection and tissue healing. An example of the rehabilitation protocol following THA in the perioperative in-patient phase is reported in Table 2. In successive phases, personalized exercise programs including gait training, range-of-motion exercises, balance training, resistance training, cardiorespiratory exercise, and flexibility are delivered to patients to restore hip function, prevent falls [74, 88, 89], and facilitate the resumption of recreational activity [99].

**Table 2:** Example of a rehabilitation protocol and goals for patients following THA by the DDA

	Postoperative day 1	Postoperative day 2	Postoperative day 3	Postoperative day 4
AM	Even in the presence of drainage - Instructions to patient - Mobilization of TC joint (ankle pump) - Exercises to increase hip ROM and muscle strength - Lateral position - Standing from seated position - Standing with partial weight using aids (walker)	- Mobilization of TC joint (ankle pump) - Exercises to increase hip ROM and muscle strength - Lateral position - Standing from seated position - Standing and walking with partial load using aids (walker/crutches)	- Exercises to increase hip ROM and muscle strength - Standing from seated position - Bed–chair transfer - Standing and walking with partial load using aids (walker/crutches)	- Exercises to increase hip ROM and muscle strength - Bed–chair transfer - Standing and walking with partial load using aids (walker/crutches) - Autonomous in ADL - Stairs
PM	- Mobilization of TC joint (ankle pump) - Exercises to increase hip ROM and muscle strength - Lateral position - Standing from seated position - Standing and walking with partial load using aids (walker)	- Mobilization of TC joint (ankle pump) - Exercises to increase hip ROM and muscle strength - Lateral position - Standing from seated position - Bed–chair transfer - Standing and walking with partial load using aids (walker/crutches)	- Exercises to increase hip ROM and muscle strength - Standing from seated position - Bed–chair transfer - Standing and walking with partial load using aids (walker/crutches) - Stairs	- Exercises to increase hip ROM and muscle strength - Bed–chair transfer - Standing and walking with partial load using aids (walker/crutches) - Autonomous in ADL - Stairs

*TC* talocrural,* ROM* range of motion,* ADL*  activities of daily living

### Rehabilitation progression

Progressive muscle strengthening is necessary to recover proper hip function after THA**.** Notably, the DAA does not require muscle detachment, leading to reduced tissue damage and improved strength compared to the LA in the first postoperative week [65], although the tensor fasciae latae, rectus femoris, sartorius, and gluteus medius undergo stretching during the procedure. Muscle damage from the surgery combined with prior muscle weakness, which is commonly observed in the affected limb (hip and knee extensors and flexors) of individuals with late-stage osteoarthritis (OA), are a focus of rehabilitation programs [90, 91]. It is recommended to focus on improving muscle strength in these groups by 30–40% to reach a healthy level [92] and ensure gait safety. Several protocols are available and may vary among institutions and surgeons; a general physiotherapy program is structured to optimize recovery, improve mobility, and ensure a successful outcome as follows:

1. Preoperative education and assessment:
  Educate the patient about the procedure, expected outcomes, and postoperative rehabilitation. Neither forced extension nor external rotation or twisting while standing are advised during the first 6 weeks after surgery.
  Perform a thorough preoperative assessment, including hip and knee ROM, strength, quality of gait, global functional status, and pain levels at rest and during activity.
2. Immediate postoperative phase (day 1 and day 4):
  See Table 2
3. Early postoperative phase (day 4 to week 2):
  Pain and swelling management: continue.
  ROM exercises: progress from passive to active-assisted and active ROM exercises for the hip joint, including flexion, abduction, adduction, and rotation.
  Strengthening exercises: begin gentle strengthening exercises for the hip abductors, quadriceps, hamstrings, and gluteal muscles to improve stability and function.
  Gait training: focus on improving the gait pattern, stride length, and symmetry during walking. Gradually reduce the reliance on assistive devices, based on individual progress.
  Functional activities: introduce functional tasks such as sit-to-stand, stair climbing, and getting in/out of a car to simulate real-life scenarios.
  Education and home exercise program: educate the patient on joint protection strategies, and provide them with a structured exercise program for at-home self-management.
4. Intermediate rehabilitation phase (week 2 to week 6):
  Progressive strengthening: increase the intensity and resistance of strengthening exercises for the lower extremities, incorporating resistance bands, weights, and functional movements.
  Balance and proprioception training: exercises to improve balance, proprioception, and coordination, which are essential for stability during daily activities.
5. Advanced rehabilitation phase (week 6 to week 12 and beyond):
  Advanced strengthening and conditioning: progress to more advanced strengthening exercises targeting specific muscle groups and functional movements relevant to the patient's goals.
  Endurance training: incorporate cardiovascular exercises such as stationary biking, swimming, or walking to improve overall endurance.
  Return to activities: collaborate with the patient to establish goals for returning to work, hobbies, sports, or recreational activities.
  Long-term maintenance: emphasize the importance of supporting an active lifestyle, regular exercise, and periodic follow-ups to check joint health and function.

The timing and progression of recovery depend on the patient’s characteristics (age and physical, functional, and clinical condition) and the orthopedic surgeon’s postoperative recommendations. Compared to lateral approaches, short-term rehabilitative goals can be achieved earlier through the use of appropriate perioperative analgesics, minimally invasive techniques, and prosthetic constructs that preserve bone stock and by facilitating fast-track protocols. In selected patients, physiotherapy and ambulation with aids can start 4–6 h postsurgery. The return to recreational activities after THA is dependent on several factors, including the type of activity (low- vs high-impact activity), the patient’s profile, and the surgeon’s opinion. In a recent study by Mead and Bugbee [93], most patients did not report any limitations when returning to sports after THA for primary or posttraumatic osteoarthritis or among those who returned to activity, In the DAA group, 71% tried their main presurgery sport, compared with 53% in the PLA group. Walking and cycling were the most common low-impact recreational activities, while exercise classes and weightlifting were the most common high-impact ones. In a larger study evaluating the activity and participation of patients following THA by either the DAA or PLA, younger and healthier subjects in the DAA group reported better functional recovery, greater fulfillment of expectations for surgery outcomes, and a faster return to work compared with PLA patients [94].

As recently emphasized by Konnyu et al. [95], the rehabilitation interventions for first total THA are too varied (in program content and intensity, personnel, setting, and progression) to determine if specific factors influenced the outcomes. Further studies are needed to evaluate interventions and standardize protocols and outcomes.

### Delivery-of-care models

Rehabilitation following DAA THA is an underresearched area in orthopedics. There is little to guide clinical decisions to optimize outcomes. Much of this research is based on the risk of adverse events and complications. The evolution of this procedure led to a similar rehabilitation approach to LA. In a large study by Van Den Eeden et al., 378 subjects who were randomized into a fast-track program (24 h to discharge) were compared to patients who underwent the usual rehabilitation protocol. Subjects who were provided with expedited care were discharged without any significant difference in patient satisfaction, pain, dislocation, or reoperation rate [64]. Most studies that look at these comparative outcomes limit their populations to those without known risk factors (BMI > 30, elderly, etc.). In a study by Oberfeld which included these high-risk subjects, no difference in complications or adverse events was found when those subjects were compared to low-risk patients in the DAA THA population [12].

Research findings support the use of fast-track programs in those with DAA THA to reduce the burden from the hospitalization of these patients, as there was no significant increase in risks when discharging to skilled-nursing facilities or the patient’s home. Further research is needed to assess the long-term results of these fast-track programs, and these are currently being investigated by several clinical trials [96, 97].

The medium in which rehabilitation is provided is another area of delivery of care that is currently under investigation and may have profound impacts on the long-term outcomes and cost associated with DAA THA. Some countries provide lump payments for certain procedures, and THA is one of them. These systemic changes, as well as the COVID-19 pandemic, required healthcare providers to administer care through telehealth. Hofman et al. found that there was a significant increase in the use of telehealth during the pandemic for a variety of orthopedic and trauma conditions, and they found no increase in complications and some advantages for patient satisfaction [98]. Rao et al. explored this further in the DAA THA population and found that patients preferred the telehealth option of rehabilitation through videos, exercises, and other resources as compared to in-person rehabilitation [13]. After 6 weeks of either telehealth or in-person rehabilitation, subjects were able to switch groups. Seventeen percent of the telehealth group moved to the in-person group and 63% of the in-person group moved to the telehealth group [13]. Patients prefer the telehealth option, but more research is needed to help identify the long-term outcomes and the patient profiles that may benefit more from the in-person rehabilitation. Rao et al. found that those who experienced more pain and lower function preoperatively were more likely to choose the in-person model of care.

There is limited evidence currently to support a specific level of intensity or approach to maximize these early gains from the anterior surgical approach. The early mobility gains and lower amounts of pain provide an environment that rehabilitation has yet to capitalize on to improve patient outcomes and reduce costs. This lack of evidence requires further exploration to better inform healthcare providers in the management of this successful procedure. Rehabilitation after DAA THA should start 3–4 h after surgery in the hospital and eventually transition from in-person rehabilitation to telehealth. The timing and identification of subpopulations needs further research to help guide the care pathways for patients receiving DAA THA.

## Conclusion

In conclusion, our review underscores the imperative for ongoing research in the realm of rehabilitation after DAA THA. While current literature offers limited guidance, emerging studies suggest that accelerated care pathways can yield comparable outcomes in terms of patient satisfaction, pain control, and complication rates. The advancement of surgical techniques and implant technologies has further bolstered the effectiveness of this approach across diverse patient cohorts. Looking ahead, the continuous exploration and refinement of rehabilitation protocols customized to the unique requirements of individuals undergoing minimally invasive THA will be pivotal to optimizing results and elevating postoperative recovery standards in the fields of surgical and rehabilitation medicine.

## Data Availability

Not applicable.
